# Koumiss, or Araby Milk

**Published:** 1884-07

**Authors:** 


### KOUMISS, OR ARABY MILK.

IS fermented milk, made in Asia from the milk of mares or camels. When distilled, koumiss yields an alcoholic liquor. In this country koumiss is made from cow's milk in the following manner : To one pint of fresh milk add one yeast cake, or enough to produce fermentation. Let it stand from 12 to 18 hours in a place where the temperature is not too warm or too cold, but just adapted to induce slow fermentation. Stir occasionally with a spoon. The first koumiss made will be unfit for use on account of its tasting of the yeast ; therefore take one gill of this koumiss and add to a pint of fresh milk, leaving for 12 or 18 hours as before. Continue this process for four or five days, but on the fifth day add a gill of the koumiss to one quart of milk ; this quart when fermented will be fit for use, and is about the quantity required for food of one person for one day. Always be careful to save a gill of koumiss each day as yeast or seed for the new quart.

When complete, koumiss resembles thick sour milk, but the difference seems to be that in the process of souring the milk is decomposed into carbonic acid gas and acetic and lactic acid ; whereas koumiss by yeast fermentation is split up into carbonic acid gas and alcohol, rendering it easy of digestion ; on the same principle that sweet yeast-fermented bread is easier of digestion than bread that has turned sour in rising.

### USES OF KOUMISS.

In diseases depending on derangement of the digestive organs koumiss is a most valuable nutrient. It contains all the elements necessary to the complete nourishment of the system, and is easily digested by stomachs that will hardly tolerate ordinary foods. Under the exclusive use of koumiss the most rebellious stomach becomes quiet in a few days, and does its work easily. The digestive organs have an opportunity to rest from severe labor, and when the stomach is thus relieved there is always more or less improvement in the symptoms of other diseases, whatever their character.

### HOW TO USE KOUMISS.

Break up bread enough to fill a pint bowl ; pour the koumiss over it, and then sprinkle on this one or two teaspoonfuls of granulated sugar, and eat the same as bread and milk. The patient usually becomes very fond of this, and has little desire for other food,—in fact, the bread and koumiss should be eaten three times a day, and no other foods taken for the first ten days, and even then only those that are easily digested. As a rule, acid foods and drinks should be abstained from. The object of this treatment is to give the system rest during a time sufficient for the powers of nature to effect a cure. The great obstacle to the cure of all diseases is the difficulty or impossibility of keeping the diseased parts quiet long enough for Nature to make her repairs.

Koumiss is not fit for use after it has stood twenty-four hours, particularly in warm weather. There is a koumiss sold in bottles. In our opinion such koumiss is unfit for use by invalids, since it contains alcohol, added to prevent further fermentation ; and experience has proved that relief that is afforded by alcoholic stimulants is paid for later with added penalties. It may be remarked that fresh milk is the natural food for infants, but it does not agree with the adult stomach when digestion is impaired.

